# Fatty acid composition of vegetable oil blend and *in vitro* effects of pharmacotherapeutical skin care applications

**DOI:** 10.1590/1414-431X20188209

**Published:** 2019-02-14

**Authors:** M. Guidoni, M.M. de Christo Scherer, M.M. Figueira, E.F.P. Schmitt, L.C. de Almeida, R. Scherer, S. Bogusz, M. Fronza

**Affiliations:** 1 Programa de Pós-Graduação em Ciências Farmacêuticas, Laboratório de Produtos Naturais, Universidade Vila Velha, Vila Velha, ES, Brasil; 2 Instituto de Química de São Carlos, Universidade de São Paulo, São Carlos, SP, Brasil

**Keywords:** Vegetable oil blend, Skin care, Fatty acids, Anti-inflammatory, Nitric oxide, Scratch assay

## Abstract

Vegetable oils have been used for a plethora of health benefits by their incorporation in foods, cosmetics, and pharmaceutical products, especially those intended for skin care. This study aimed to investigate the cutaneous benefits of a vegetable oil blend (VOB) formulation and its fatty acid composition. The anti-inflammatory activity was studied in macrophages of RAW 264.7 cells by investigating the release of nitric oxide (NO), superoxide anion generation (O_2_^-^), tumor necrosis factor-alpha (TNF-α), and interleukin 6 (IL-6). ABTS cation radical scavenging capacity assay, ferric reducing antioxidant potential (FRAP), 2,2-diphenyl-1-picrylhydrazyl (DPPH), and NO free radical scavenging assays were used to evaluate the antioxidant activity. VOB was tested for its ability to stimulate fibroblast proliferation and migration using the scratch assay, and antibacterial activity by the microdilution test. The fatty acid profile of a freshly prepared VOB formulation was determined by gas chromatography before and after accelerated stability testing. Chemical composition of VOB revealed the presence of oleic acid (C18:1n-9; 63.3%), linoleic acid (C18:2n-6; 4.7%), and linolenic acid (C18:3n-6; 5.1%) as major mono- and polyunsaturated fatty acids. No changes in the organoleptic characteristics and fatty acid composition were observed after the accelerated stability test. VOB 100 µg/mL reduced the healing time by increasing the total number of cells in the wounded area by 43.0±5.1% compared to the negative control group. VOB also suppressed the pro-inflammatory TNF-α and IL-6 cytokines, and NO and O_2_^-^ production in lipopolysaccharide-stimulated macrophage cells. In conclusion, the VOB formulation contributed to the improvement of current therapeutic strategies for cutaneous applications in skin care.

## Introduction

Due to a widespread lifestyle shift caused by a multitude of factors in the last years, there is a growing demand for natural products. The world is considered a rich source of natural products. Vegetable oils, which are obtained from many renewable resources, are getting more attention than ever due to the numerous health benefits. Moreover, vegetable oils have attracted a great interest for the development of natural and eco-friendly cosmetics (1,2). The medicinal value is based on the bioactive fatty acids constituents that have multiple skin benefits and may produce definite physiological action on the human body (3–5).

Vegetable oils are composed primarily by triacylglycerols and a sparse amount of diacylglycerols and monoacylglycerols. They also contain phospholipids, free sterols, tocopherols, and tocotrienols, triterpene alcohols, hydrocarbons, and fat-soluble vitamins in small amounts. Fatty acids composition of vegetable oils is classified according to the presence or absence of double bonds as saturated fatty acids (without double bonds), monounsaturated fatty acids (with one double bond), and polyunsaturated fatty acids (with more than two double bonds). The chain length and degree of unsaturation may have great influence on the chemical biological properties of these compounds. In addition, genetic and environmental factors can determine the proportions of saturated and unsaturated fatty acids present in vegetable oils (1,6).

Wound healing is a natural process in which the body responds to an injury to protect itself from the outer environment while skin repair occurs simultaneously (7). Wound healing runs in three basic phases: inflammatory, proliferative, and maturation (8). After skin injury, the wound healing process begins immediately with clotting formation at the wound site by platelet aggregation and vasoconstriction. The pro-inflammatory cytokines and chemokines including interleukin-6 (IL-6) and tumor necrosis factor (TNF-α) are released to activate inflammatory cells (7,9). Neutrophils, macrophages, and fibroblasts are infiltrated to the wound site. Then, nitric oxide, oxygen free radicals, and matrix metalloproteinase are generated to prepare for the proliferation phase (10). The proliferation phase involves epithelialization and angiogenesis in which transforming growth factors and epidermal growth factor are important factors for stimulating proliferation, migration, and differentiation of fibroblasts and keratinocytes, forming extracellular matrix and collagen, and finally, tissue remodeling (9).

Vegetable oils are frequently used to treat wounds, mainly in developing countries. The most abundant fatty acids responsible for their therapeutic effect are oleic, linoleic, and linolenic acids (11–13). The non-saponifiable lipids are usually responsible for the anti-inflammatory and antioxidant effects of vegetable oils. Fatty acids are necessary for the maintenance of epidermal integrity and the water barrier of the skin. They are metabolic precursors of arachidonic acid and prostaglandins in the epidermis and important for regulation of cell division and epidermis differentiation (2,14).

Therefore, our proposal was to develop a VOB formulation with vegetable oils of varying origin and fatty acid composition and to evaluate the therapeutic potential related to its *in vitro* antioxidant, anti-inflammatory, and antibacterial effects, and its capability to stimulate the proliferation and migration of fibroblasts.

## Material and Methods

### Chemicals

TNF-α and IL-6 ELISA kits were from eBioscience (USA). All other reagents were obtained from Sigma-Aldrich (USA). All solvents were of analytical grade and obtained from various commercial sources. The vegetable oils were purchased from SM Produtos Farmacêuticos (Brazil).

### Cell lines

Mouse macrophages RAW 264.7 (American Type Culture Collection, ATCC^®^ TIB-71™) and murine fibroblasts (L929 cell line, ATCC^®^-CCL1™) (Cell Line Service, Brazil) were maintained in Dulbecco's modified Eagle's medium (DMEM) supplemented with 10% fetal bovine serum (FBS), 100 IU/mL penicillin, and 100 µg/mL streptomycin, at 37°C, in a humidified atmosphere containing 5% CO_2_ (all Sigma, USA).

### Preparation of the vegetable oil blend

The vegetable oil blend (VOB) was prepared by the direct mixture of flaxseed oil (15%), blackcurrant oil (10%), olive oil (20%), rosehip oil (10%), macadamia oil (15%), and sunflower oil (30%). The VOB was stored in an amber glass bottle in the absence of light and moisture at room temperature.

### VOB fatty acid profile

VOB fatty acid methyl esters (FAME) were prepared by methylation with boron trifluoride (BF3) in methanol according to Joseph and Ackman (15). The FAME composition was determined by gas chromatography (GC-2014, Shimadzu, Japan), coupled with a flame ionization detector (FID). Fatty acids were identified by comparing the retention time using authentic standards of FAME (GLC85 reference standard, Nu-Chek-Prep, USA). The internal standard used was methyl tricosanoate (C23:0 reference standard, Nu-Chek-Prep). FAME were separated on a capillary column DB-5 (30 m × 0.25 mm I.D. × 0.25 μm) (Agilent Technologies, USA). Nitrogen was used as a carrier gas at 0.6 mL/min. The chromatographic conditions were injector 250°C, split 1:50, injection volume 1 µL; oven: 100°C for 0.5 min, followed by an increment of 3°C/min to 260°C; FID was maintained at 280°C.

### VOB stability testing

To estimate the stability of the VOB and the expiration date, accelerated stability testing was performed according to the Brazilian Health Regulatory Agency (16). The VOB sample was stored in a transparent, neutral glass bottle with a cover that guaranteed a proper seal avoiding loss of gases and evaporation to the medium. Then, the freshly prepared VOB was submitted to heating in an oven at 45±2°C, alternating with cooling in the refrigerator at 5±2°C, with cycles of 24 h each over 4 weeks. Organoleptic characteristics (color, odor, and appearance) and FAME profile were evaluated before and after the accelerated stability.

### DPPH free radical scavenging assay

The DPPH scavenging activity of VOB (1–2000 µg/mL) was evaluated from the bleaching of the purple methanol solution of free radical DPPH according to Benevides et al. (17). The antioxidant activity is reported as IC_50_ value (µg/mL) obtained from three independent experiments.

### Nitric oxide free radical scavenging assay

The compound sodium nitroprusside is known to spontaneously generate nitric oxide, which interacts with oxygen to produce nitrite ions that can be estimated using the Griess reagent (17,19). Briefly, the reaction mixture containing sodium nitroprusside in phosphate-buffered saline with or without the VOB was incubated at room temperature for 30 min. Then, 150 μL of incubated solution was mixed with 150 µL of Griess reagent and the absorbance of chromophore formed was measured at 540 nm in an ELISA plate reader (SpectraMAX 190, Molecular Devices, USA). The results are reported as IC_50_ value (µgmL). Experiments were carried out at least in triplicate.

### Ferric reducing antioxidant potential assay (FRAP)

Antioxidant capability of VOB was estimated as described by Pulido et al. (18) with modifications by Benevides et al. (17). FRAP reagent was mixed with VOB or ethanol (for the reagent blank), incubated at room temperature for 10 min, and then the absorbance was measured at 595 nm using a microplate reader (SpectraMax 190, Molecular Devices). The results are reported as IC_50_ value (µg/mL). Experiments were carried out at least in triplicate.

### ABTS cation radical scavenging assay

The antioxidant activity was determined according to Re et al. (20). In 96-well microplates, 270 µL of ABTS radical cation was mixed with 30 µL of VOB (1–2000 µg/mL) (ethanol for blank) and allowed to react in the dark during 10 min. Absorbance was measured at 734 nm using a microplate reader (SpectraMax 190, Molecular Devices). The results are reported as IC_50_ value (µg/mL). Experiments were carried out at least in triplicate.

### *In vitro* cytotoxicity

Cellular viability was measured using the MTT assay according to Marques et al. (21). Macrophages RAW 264.7 and L929 fibroblast cells were incubated for 24 h in the presence or absence of VOB from 1–1000 µg/mL concentration. Experiments were carried out at least in triplicate and results are reported as percentage of viable cells.

### *In vitro* wound healing (scratch) assay

The *in vitro* scratch wound assay was carried out as previously described (24) with modifications (25). Briefly, fibroblasts were cultured to nearly confluent cell monolayers and then an artificial linear wound was made. Then, the monolayers were treated for 16 h with different concentrations of VOB (1–200 µg/mL). VOB was diluted in dimethyl sulfoxide (DMSO) and thereafter diluted in growth medium at the desired concentration. DMSO concentration in the wells was kept under 0.5% concentration. Platelet derived growth factor (PDGF) was used as positive control. After incubation, cells were fixed and stained with 2-(4-amidinophenyl)-1-indole-6-carboxamidine (DAPI) and the cellular migration into the wounded area was quantified using CellC^®^ software (Finland). Results are reported as percentage of cells that migrated and/or proliferated into the injured area compared to the untreated control group.

### Nitric oxide analysis in the supernatant of macrophage cell culture

NO production was determined by measuring the amount of nitrite in lipopolysaccharide (LPS)-stimulated macrophages supernatant according to the Griess reaction (19) with minor modifications (21,22). RAW 264.7 cells were treated with LPS (1 µg/mL) with or without VOB (10–200 µg/mL) for 24 h. Next, the culture supernatant was mixed with Griess reagent (1:1) and incubated for 10 min. Absorbance at 540 nm was measured in an ELISA plate reader (SpectraMax 190; Molecular Devices) and the inhibitory rates were calculated using a standard calibration curve prepared with sodium nitrite compared to LPS-stimulated control group.

### Quantitative colorimetric nitroblue tetrazolium assay

The determination of intracellular superoxide anion production was evaluated in LPS-activated murine macrophages RAW 264.7 (21,23). Briefly, RAW 264.7 cells were seeded at a density of 2×10^5^ cell/mL in 96-well plates and cultured in a 37°C humidified incubator with 5% CO_2_ in air for 24 h. Then, cells were stimulated with 1 μg/mL LPS in the presence or absence of increasing concentrations (10–200 µg/mL) of VOB for 24 h. After incubation, the supernatant was removed and 100 µL nitroblue tetrazolium (NBT) (1 mg/mL) was added to each well. The cells were washed with methanol and dried for 20 min at 37°C. After incubation of 2 h, the formazan crystals formed were dissolved with dimethyl sulfoxide (DMSO) and potassium hydroxide (KOH). Absorbance was measured at 620 nm, using a microplate reader (Multi-Mode Microplate Reader, FilterMax F5, Molecular Devices). The experiments were carried out at least in triplicate.

### Measurement of cytokines

Quantification of TNF-α and IL-6 production in the supernatant of LPS-activated murine macrophages RAW 264.7 after VOB exposure was determined by enzyme-linked immunosorbent assay (ELISA) using specific antibodies (purified and biotinylated) and cytokine standards, according to the manufacturer's instructions (eBioscience, USA). Absorbance was measured at 450 nm in a microplate reader (Multi-Mode Microplate Reader, FilterMax F5, Molecular Devices). Cytokine levels are reported in pg and sensitivities were >10 pg/mL.

### Antibacterial activity

The minimum inhibitory concentrations (MICs) of VOB were determined against the Gram-positive bacteria *Staphylococcus aureus* (ATCC 25923) and the Gram-negative bacteria *Escherichia coli* (ATCC 8739) by the standard NCCL method (NCCL, 2008), in a 96-well microtiter plate according to Benevides et al. (17). Different concentrations of VOB ranging from 62.5 to 2000.0 µg/mL were tested. The experiments were carried out at least in triplicate.

### Statistical analysis

Data were analyzed by ANOVA and the post-hoc Tukey test, using GraphPad software (USA). Kolmogorov-Smirnov test was used to confirm the normality of data. All data are reported as the means±SE or SD, and P<0.05 values indicated statistically significant differences.

## Results

### Fatty acid composition and stability of VOB

Characterizations in the freshly prepared VOB and after the stability testing are reported as the percentage of total methyl esters and were analyzed by GC-FID. The VOB FAME composition is presented in Table 1 showing the presence of monounsaturated fatty acid (66.14%) with oleic acid (C18:1n-9; 63.39%) as the major lipid followed by the linoleic acid (C18:2n-6; 4.79%) and linolenic acid (C18:3n-3; 5.09%) as the major polyunsaturated fatty acids (10.72%). The fatty acid profile of the sample did not significantly change after accelerated stability testing, and the characteristics of the polyunsaturated fatty acids were preserved. Moreover, no changes in color, appearance, odor, or viscosity were observed after the accelerated stability test. The results indicated that organoleptic characteristics of the VOB were markedly stable during the test period.

**Table 1.** Relative percentages of fatty acid methyl esters (FAME) in freshly prepared vegetable oil blend (VOB) and after stability testing.

FAME	Fresh VOB (%)	VOB after stability testing (%)
Saturated		
10:0	0	0.02
12:0	0.32	0.37
14:0	0.32	0.33
15:0	0.02	0.02
16:0	15.05	15.33
17:0	0.12	0.12
18:0	5.98	5.99
20:0	0.89	0.88
22:0	0.66	0.06
Total	23.39	23.71
Monounsaturated		
16:01	2.62	2.76
18:1n-9	63.39	63.32
20:1n-9	0.08	0.08
22:1n-9	0.05	0.06
Total	66.14	66.23
Polyunsatured		
18:2n-6	4.79	4.00
18:3n-6	5.09	4.24
20:4n-6	0.06	0.06
20:2n-6	0.08	0.05
20:3n-6	0.68	0.67
20:3n-3	0.02	0.02
Total	10.72	9.05

### VOB *in vitro* anti-oxidative effect

Natural antioxidants play an important role in providing stability to vegetable oils hampering their oxidation. Antioxidant activity should not be concluded based on a single antioxidant test model, therefore, antioxidant activity of VOB was evaluated using four different chemical assays and the results are presented in Table 2. VOB exhibited only slight antioxidant activity (IC_50_ 233.7±1.48 µg/mL) estimated by its ability to reduce ferric iron (Fe^3+^) to ferrous iron (Fe^2+^) compared to tocopherol (IC_50_ 9.68± 2.30 µg/mL). VOB did not exhibit any scavenging activity of DPPH, ABTS, and NO radical (Table 2) up to 2000 µg/mL.

**Table 2.** Antioxidant activity of vegetable oil blend (VOB) determined by the chemical tests DPPH, ABTS, FRAP, and NO radical scavenging assay.

Sample	Antioxidant activity (IC_50_ µg/mL)			
	DPPH	FRAP	ABTS	NO
VOB	>2000.0	233.7±1.5^a^	>2000.0	>2000.0
Tocopherol	9.7±2.3	4.8±0.2^b^	6.5±0.5	

Data are reported as means±SE (n=3). Experiments were carried out at least in triplicate. Different letters in the same column correspond to significant differences (P<0.05, ANOVA and Tukey's post-hoc test).

### VOB did not exhibit *in vitro* cytotoxicity

**Figure 1.**
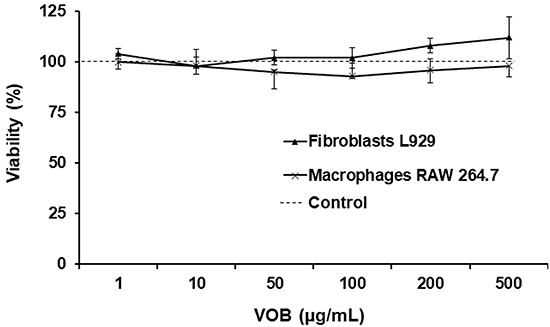
Cellular viability of fibroblasts and macrophages after 24 h exposure with 1 to 500 µg/mL vegetable oil blend (VOB) solution by the colorimetric MTT assay. Data are reported as mean±SE percentage of cellular viability compared to basal control cells of three independent experiments.

Next, the colorimetric MTT assay was performed to determine the appropriate concentration of the blend, which would not affect cellular viability. VOB was tested in non-cancerous L929 fibroblasts and murine macrophages RAW 267.7 cells. VOB did not exhibit any cytotoxic effect against fibroblasts and macrophages compared to basal control (only cell culture medium, considered as 100% viability), showing 112 and 108% viability at the highest concentration tested of 1000 µg/mL, respectively (Figure 1).

### *In vitro* cell migration/proliferation

**Figure 2.**
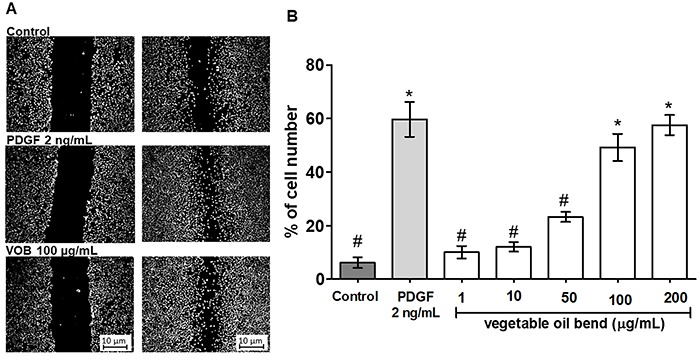
Vegetable oil blend (VOB) increased migration of fibroblasts in the scratch assay. Cells were treated with 1 to 200 µg/mL of VOB, 2 ng/mL of platelet derived growth factor (PDGF), or only with medium as control. **A**, Representative images were taken immediately after creating the wound (0 h) and after 16 h of incubation (100× magnification; bar 10 μm). **B**, Percentage of cells after 16 h in the injured area compared to the scratch area at time zero (0 h). Data are reported as the mean±SE of two independent experiments. *P<0.05 compared to control group; ^#^P<0.05 compared to the positive control group (ANOVA and the Tukey's post-hoc test).

To evaluate *in vitro* cell migration/proliferation, the scratch assay was performed using fibroblasts. After 16 h in culture with VOB at different concentrations, the results showed a positive dose-dependent enhancement in fibroblast migration/proliferation in the artificial gap, especially at 100 and 200 µg/mL VOB concentration reaching values of 49.2±5.1% and 57.5±3.7%, respectively, when compared to control cells (6.2±2.0%; P<0.05). The significant stimulatory effects of VOB were comparable to PDGF (59.4±6.8%) used as positive control (Figure 2).

### *In vitro* NO in macrophages

**Figure 3.**
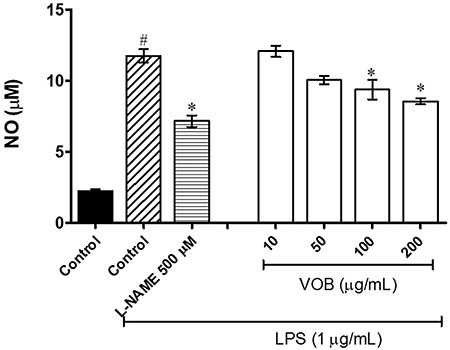
Inhibitory effects of vegetable oil blend (VOB) on nitrite production of lipopolysaccharide (LPS)-activated RAW 264.7 cells. LPS (1 µg/mL) with or without VOB (10 to 200 µg/mL) was added to cells and nitrite concentration was measured by Griess reaction assay. Data are reported as means±SE of triplicate experiments. *P<0.05 compared with the LPS-treated group. ^#^P<0.05 compared with the unstimulated control group (ANOVA and the Tukey's post-hoc test).

LPS-stimulated macrophages induced significant NO production, which was significantly blocked by VOB in a dose-dependent manner (Figure 3). Reductions of 20.1± 1.2% and 22.9±0.9% were observed in the macrophages supernatant after treatment with 100 and 200 µg/mL VOB concentrations, respectively.

### Effect of VOB on the production of superoxide anion in LPS-stimulated RAW 264.7 macrophage cells

**Figure 4.**
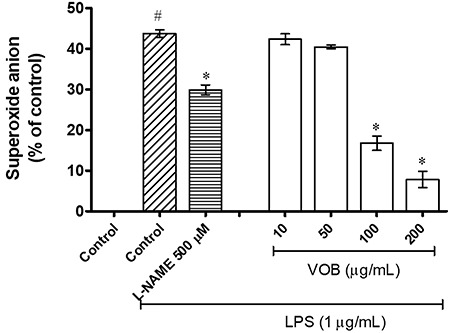
Inhibitory effects of vegetable oil blend (VOB) on intracellular superoxide anion production in lipopolysaccharide (LPS)-activated RAW 264.7 cells using the colorimetric NBT assay. Gallic acid (GA) was used as positive control. The level of superoxide anion in control cells was arbitrarily reported as zero. Data are reported as means±SE of triplicate experiments. *P<0.05 compared with LPS-treated cells. ^#^P<0.05 compared with control (ANOVA and the Tukey's post-hoc test).

The colorimetric NBT assay was used to measure the intracellular production of superoxide anion (O_2_^-^) in LPS-stimulated RAW 264.7 macrophage cells. As shown in Figure 4, O_2_^-^ production induced by LPS decreased significantly (P<0.05) after 100 and 200 µg/mL VOB was added compared to the control group.

### Determination of cytokine production

**Figure 5.**
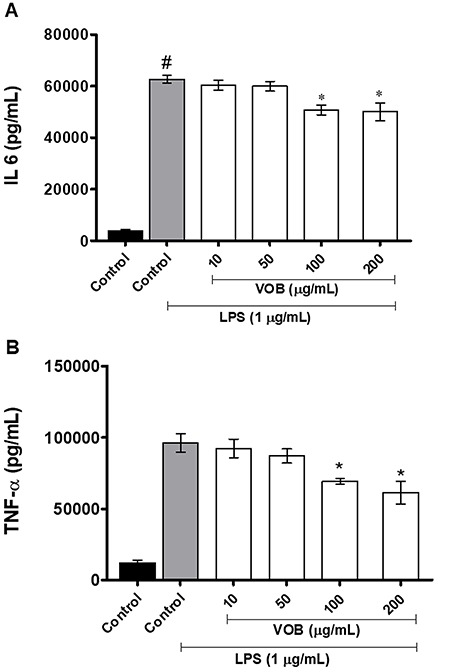
Effect of vegetable oil blend (VOB) on proinflammatory cytokine production in lipopolysaccharide (LPS)-stimulated macrophages. **A**, IL-6 and **B**, TNF-α production was measured using ELISA kits. Data are reported as means±SE of two independent experiments. *P<0.05 compared with LPS-treated cells. ^#^P<0.05 compared with control (ANOVA and the Tukey's post-hoc test).

Concerning cytokines production, the effects of VOB on LPS-induced inflammation in RAW 264.7 macrophages were evaluated by measuring the production of TNF-α and IL-6 cytokines. As observed in Figure 5, stimulation with LPS for 24 h significantly induced the release of cytokines pro-inflammatory markers (TNF-α and IL-6) indicating that an inflammatory response was induced in the macrophages. Interestingly, VOB significantly reduced the production of IL-6 and TNF-α production at 100 and 200 µg/mL.

### Antibacterial activity of VOB

Wound bacterial colonization presents a continuous challenge in the treatment of skin injuries and wound healing. Therefore, antibacterial activity of VOB was preliminarily assayed against the common Gram-positive *Staphylococcus aureus* bacteria and the Gram-negative *Escherichia coli* bacteria frequently hosted in cutaneous wounds (24). VOB exhibited only slight activity against *S. aureus* with a MIC value of 2000 µg/mL and did not exert any antibacterial activity against *E. coli* up to 2000 µg/mL.

## Discussion

In the present study, it was found that blending may be considered an economical and simple procedure to modify the fatty acid composition, enhancing the commercial application, and producing new specific products with a desired biological property, in general, at affordable prices (26). In general, natural vegetable oils have been used as topical therapy worldwide. They are easily accessible and a relatively inexpensive option for skin care including their therapeutic potential to positively influence cutaneous wound healing (12). Besides emollient properties, many natural oils possess specific compounds with antimicrobial, antioxidant, and anti-inflammatory activities. Moreover, it is possible to metabolize lipids derived from topically applied emollients and to utilize them as nutritional building blocks for the formation of a healthy and functional epidermal barrier (2,11,12).

Unique characteristics of vegetable oil blends are important when considering their use for topical skin care. Different ratios of essential fatty acids are major determinants of the repair benefits of natural oils. Oils with a higher linoleic acid to oleic acid ratio have better barrier repair potential (12). Specifically, the quantities of fatty acids with healing effects are highlighted as oleic fatty acid, linoleic acid, and linolenic acid (27). The fatty acid profile of VOB exhibited predominantly the monounsaturated fatty acid, oleic acid (63.39%), and the polyunsaturated fatty acids, linoleic acid (4.79%) and linolenic acid (5.09%). Thus, the fatty acid composition reinforces the potential therapeutic applicability for improving the natural skin-barrier function.

The fatty acids of the omega-3 family (linolenic acid) and omega-6 (linoleic acid) are of great importance for the inflammatory process, since they are not synthesized by *de novo* synthesis and are precursors of polyunsaturated fatty acids, such as eicosapentaenoic, docosahexaenoic, and arachidonic (28,29). Linoleic acid exhibited an important chemotactic role for macrophages, contributing to the autolytic debridement of the wound bed by increasing the production of metalloproteins inducing granulation and accelerating the healing process (13). In light of this, VOB also demonstrated great stimulatory effects on the proliferative and migratory activity of fibroblasts contributing, therefore, to granulation tissue formation and re-epithelialization of the skin.

The inflammatory phase of wound healing normally leads to the release of biologically active mediators and oxygen-free radicals such as hydrogen peroxide, superoxide anion, and hydroxyl anion, and an excess of these agents is well known to cause tissue damage and hamper tissue repair (9,10). In macrophage cells stimulated with LPS, known to be an endotoxin, the release of pro-inflammatory cytokines such as IL-6 and IL-1, and other inflammatory mediators such as NO (17,30) is induced. VOB was able to significantly suppress the production of these hazards of the healing process. The overproduction of NO has been reported to contribute to the pathogenesis of inflammatory diseases, including rheumatoid arthritis, atherosclerosis, pulmonary fibrosis, and unhealed wounds. Activated macrophages release into the extracellular medium several reactive oxygen species, including singlet oxygen, superoxide anion, among others. VOB has been shown to be highly effective in inhibiting the production of these radicals, especially NO and superoxide anion, and inflammatory cytokines, proving to be a good alternative as an antioxidant and anti-inflammatory agent (31,32).

Another significant problem with wounds is the high risk of infection. Therefore, the use of an antimicrobial agent during the healing process will help to reduce the risk of infection and the overall time for wound healing can be reduced significantly (28). Linoleic acid was proven able to inhibit the growth of *Staphylococcus aureus* by altering the synthesis of proteins, cell walls, nucleic acids, and cell membranes during division (13). Although VOB elicited only a discrete antibacterial action, it can be assumed that the topical use of VOB may protect cutaneous wounds from pathogenic bacteria and their harmful effects on wound healing.

In addition, chronic wounds of the skin present a painful, unsightly, and unpleasant sensory experience. VOB could be used to stimulate wound hydration, diminishing trauma during dressing changes. Besides, a hydrated wound favors the process of re-epithelialization, granulation, tissue formation, angiogenesis, fibroblast migration, collagen synthesis, and remodeling of injured tissue. Therefore, VOB should be used as a natural synergetic compound to treat skin wounds.

Altogether, this work highlights the role of a highly effective and low-cost vegetable oil blend, which may be used against inflammatory skin disorders or to treat skin injuries. In conclusion, more work should be done to increase our understanding of the mechanism by which VOB improved proliferation and migration of fibroblasts and altered proinflammatory and oxidative mediators in LPS-stimulated macrophages cells.
